# Topical anaesthesia reduces sensitivity of castration wounds in neonatal piglets

**DOI:** 10.1371/journal.pone.0187988

**Published:** 2017-11-15

**Authors:** Sabrina Lomax, Charissa Harris, Peter A. Windsor, Peter J. White

**Affiliations:** 1 Faculty of Science, School of Life and Environmental Science, The University of Sydney, Camperdown, NSW, Australia; 2 Faculty of Science, School of Veterinary Science, The University of Sydney, Camperdown, NSW, Australia; University of Bari, ITALY

## Abstract

The aim of this study was to do determine the efficacy of the topical anaesthetic Tri-Solfen® in the amelioration of the pain of castration in piglets. The trial was conducted over a three day period, and blocked across six litters with 12 piglets treated on days one and two, and 16 on day three. The piglets were randomly allocated by weight and litter to 1 of 4 treatment groups: (i) sham castration (SHAM; n = 10); (ii) surgical castration with no anaesthetic intervention (CAST; n = 10); (iii) surgical castration with post-operative topical anaesthesia (TRI; n = 10); (iv) surgical castration with a pre-operative intra-testicular lignocaine hydrochloride injection (LIG; n = 10). Wound sensitivity testing involved von Frey monofilaments of weights 4g and 300g, and an 18 gauge needle, used to stimulate the wound and surrounding skin respectively, at various pre-determined sites. Neonatal piglets receiving topical anaesthesia (Tri-Solfen®) spray into castration wounds had significantly lower wound sensitivity responses for up to 4h, compared to those castrated following intra-testicular lignocaine injection or those with no treatment. The use of topical anaesthetic is suggested as a practical and affordable method of improving piglet welfare during castration.

## Introduction

Castration of male piglets is a routine husbandry procedure commonly performed within the first few days of life in commercial piggeries globally. Extensive research to assess the effect of castration on the welfare of the piglets indicates that castration causes significant stress, pain and discomfort that can persist for up to 4 days [1–4].

Castration of piglets is usually performed without anaesthesia or analgesia, presumably because the anaesthetic techniques commonly used for comparable surgeries in human and veterinary medicine (general anaesthesia or sedation, local anaesthetic infiltration and / or local or regional nerve blockade) are either too complex, costly and time consuming to be practical or affordable for use on-farm. A number of publications have described the effective use of analgesic and anaesthetic interventions to address the pain associated with piglet husbandry procedures [5–9] although these options may be considered neither practical nor affordable for large scale production systems.

The European Union (EU) legislated that as of January 2012, surgical castration of pigs must utilize prolonged local anaesthesia and/or analgesia, with the aim of removing the practice altogether by 2018. Whilst cost-benefit analyses conducted in relation to this legislation indicate that general and local anaesthesia strategies are beneficial, in other jurisdictions such as Australia the costs of lsuch procedures are considered excessive, particularly where veterinarians are required by law to administer restricted drugs such as lignocaine [10].

Tri-Solfen® (Bayer Animal Health, Pymble, Australia) is a commercially available topical anesthetic spray-on wound dressing with haemostatic and antiseptic properties, registered in Australia for the alleviation of pain of castration in sheep and cattle. It contains lignocaine (40.6 g/L), bupivacaine (4.5 g/L), adrenalin (24.8 mg/L) and cetrimide (5.0 g/L) in a gel base, and has been reported to be effective in ameliorating wound pain and improving healing during mulesing, castration and tail docking in sheep and castration in calves [11–14]. Due to the efficacy of this product for reducing the post surgical pain associated with castration in lambs and calves, and the similar anatomical nature of the wounds induced in porcine castration, we studied the efficacy of Tri-Solfen® in the amelioration of the pain of castration in piglets.

## Materials and methods

All experimental protocols were approved by The University of Sydney Animal Ethics Committee (AEC Approval No. 742).

### Animals

The trial was performed using 40 Landrace x Large white piglets sourced from a commercial herd at The University of Sydney Farms near Cobbitty, NSW. Piglets were from mixed litters and aged 3–5 days old when castrated. The piglets were housed in farrowing stalls with the sow and their littermates, before and upon completion of the trial. Piglets were removed for the procedure and subsequent wound sensitivity testing, and retained in large crates containing a sawdust floor cover for the 5 hour duration of the study, with an overhead heat lamp providing warmth. The piglets were returned to their sows on conclusion of the observation period.

The trial was conducted over a three day period using piglets from six litters with 12 piglets treated on days one and two, and 16 on day three. On each day, piglets were weighed and numbered 1–12 (day one and day two) or 1–16 (day three) with marker crayon for identification.

### Treatments

The piglets were randomly allocated by weight and litter to 1 of 4 treatment groups: (i) sham castration (SHAM; *n = 10*); (ii) surgical castration with no anaesthetic intervention (CAST; *n = 10*); (iii) surgical castration with post-operative topical anaesthesia (TRI; *n = 10*); (iv) surgical castration with a pre-operative (5mins) intra-testicular lignocaine hydrochloride (1ml per testis) injection (LIG; *n = 10*).

Castration was performed by a single trained technician. Piglets were removed from the crate and restrained by their hind limbs in a vertical position with the head down. The scrotum was exposed and each scrotal sac and tunica received a small (1.5cm) incision with a sterile scalpel to express the testes. The exposed testes were then excised following incision of the spermatic cord. The whole procedure including TRI treatment, took less than 1 minute per animal. Sham-castrated piglets were handled and restrained similarly to the castrates, with the scrotum and testes manipulated, but no incision made. Piglets in the TRI group received 1mL of Tri-Solfen® spray to each scrotal incision upon exposure of the testis using a 1mL pipette to apply 0.5mL to the cut skin edge and spermatic cord prior to excision. The pipette end was then inserted along the spermatic cord inside the wound to apply a further 0.5mL to ensure that all retracted tissue was coated (12).

### Wound sensitivity testing (WST)

WST involved von Frey monofilaments of weights 4g and 300g, and an 18 gauge needle, used to stimulate the wound and surrounding skin respectively, at various pre-determined sites. These procedures were applied as per previous studies [11,12,14]. Animals were tested immediately prior to treatment and then at 1 min following treatment. Further testing was then conducted at every 30 minutes up to four hours post treatment with animals restrained in the same position used for the castration procedure. The presence or absence of skin anaesthesia was determined by recording the response to a superficial skin pinprick with an 18G needle [15–17].

Wound sensitivity was measured at 5 locations, presenting from left to right: left cut skin edge, left wound body, the skin in the middle of the incisions (Middle), right wound body and right cut skin edge. Site and von Frey weight /needle were randomized between each measurement and piglet to avoid confounding of results Involuntary motor responses were scored using an adjusted customized numerical rating scale [11, 12, 14]. Nociceptive response was graded on a scale of severity from 0 to 3, including local responses at the wound site and central responses: (Score 0 = no motor response, no discernible movement or reaction; Score 1 = mild response including minor subcutaneous muscle twitches, minor vocalization; Score 2 = moderate response including partial withdrawal reflex or startle response with minor motor movement of the whole head; moderate vocalization; Score 3 = severe response including full withdrawal reflex or full startle response with major head jerk and vocalization). All observations were made by the same experienced observer.

### Statistical analysis

For the analysis, body weight was coded into three categories (WeightCat): (1) 0.9–2.28 kg; (2) 2.3–3.38 kg; and (3) 3.3–4.7 kg. Score data was analysed using ordinal logistic regression in AsREML® (Version 16, VSNI, UK). Treatment, time, WeightCat, stimulation type (Stim) and site were included as fixed effects, with piglet and litter as the random effects. Z values were determined using logit and SED arrays, and P-values calculated. Data is presented as cumulative odds ratios with the statistical probabilities of piglets in each treatment group displaying a response score of Y = 0, 1, 2 and 3. In effect, these probabilities are the averages of the scores from the 2 von Frey weights and the 18g needle. For all statistical calculations, P values < 0.05 were considered statistically significant.

## Results and discussion

### Weight

**Fig 1:**
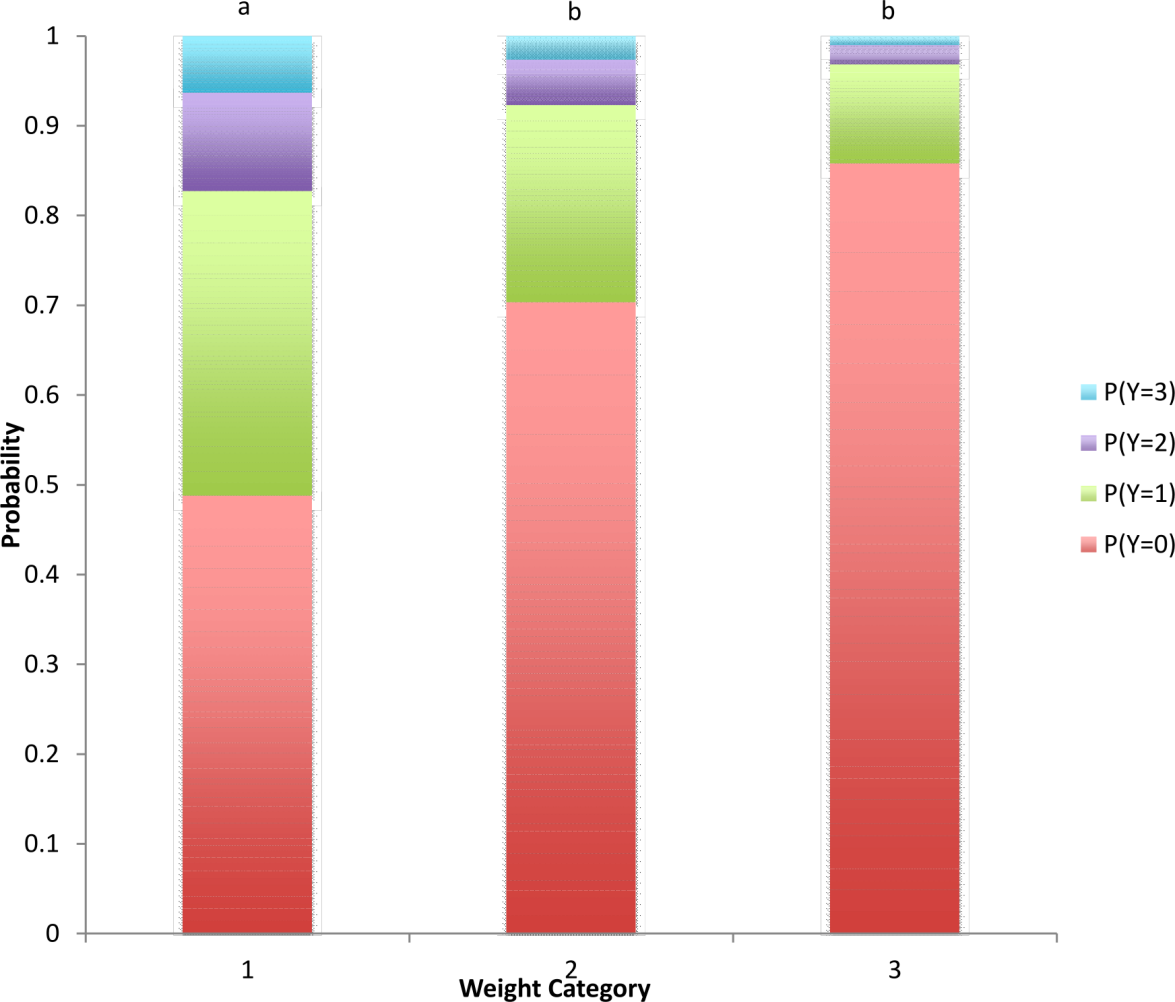
Probability of piglets of different weight categories displaying responses (Y; 0 = no response, 1 = minor, 2 = moderate, 3 = severe) to stimulation. Results combine the effect of 4g and 300g von Frey monofilaments and 18g needle for all time points. a,b Weight groups not sharing a common letter are significantly different (P < 0.05).

Mean piglet weight was 2.7 ± 0.45kg. There was a significant effect of weight on piglet response (P = 0.004). Smaller piglets (WeightCat 1) had the greatest probability of more severe response scores, with the larger piglets (WeightCat 3) least likely to respond (Fig 1).

### Treatment x Stim

**Fig 2:**
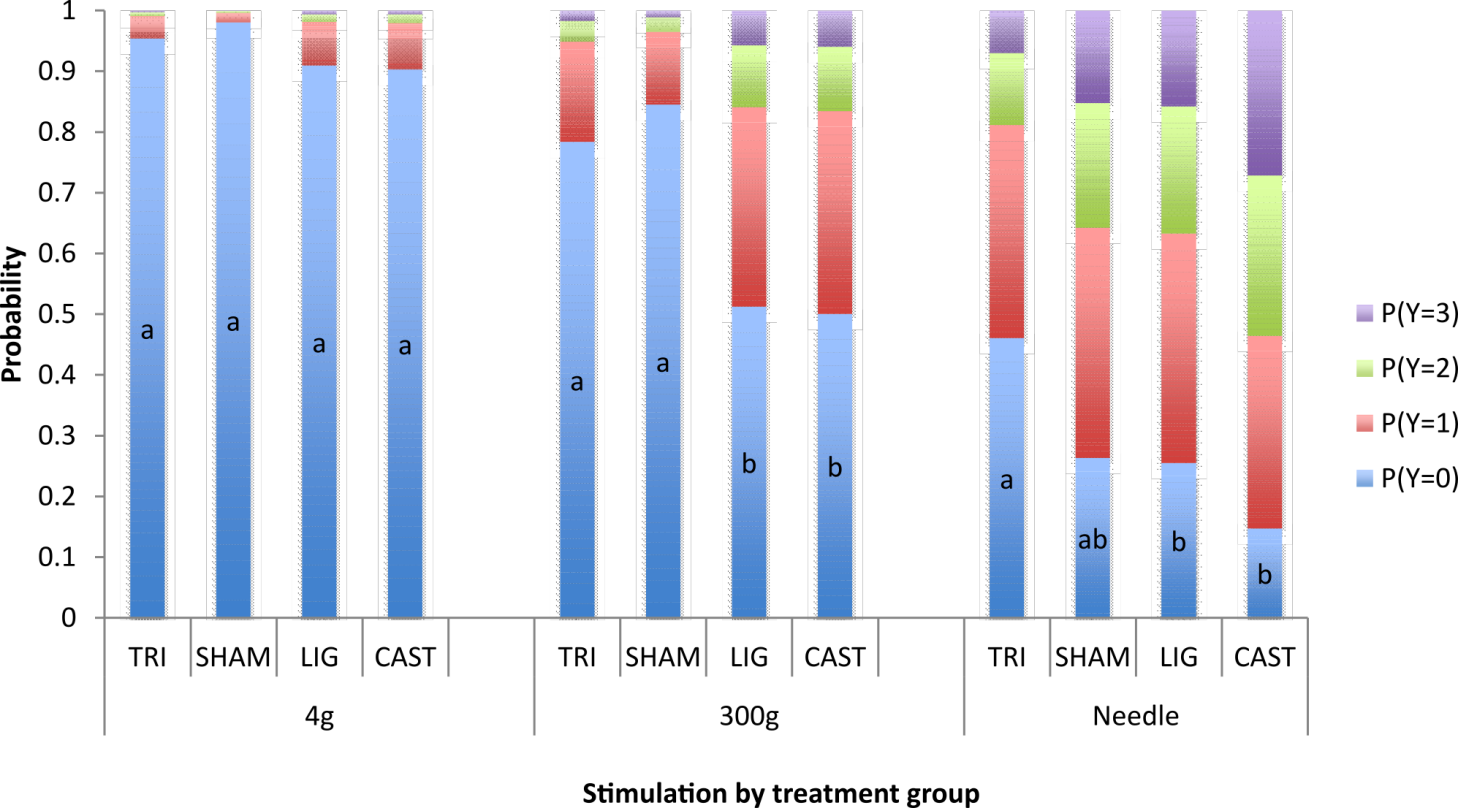
Probability of piglets of different treatment groups displaying responses (Y; 0 = no response, 1 = minor, 2 = moderate, 3 = severe) to stimulation using 4g and 300g von Frey monofilaments and 18g needle. Results combine the effect for all time points. a,b Treatment groups not sharing a common letter are significantly different (P < 0.05).

There was a significant Treatment x Stim interaction (P < 0.001). Severity of response increased significantly with weight of stimulation from the 4g von Frey to the 18G needle in all treatment groups (Fig 2). There was no effect of treatment on response to 4g stimulation. The response of TRI treated piglets and SHAM piglets to 300g stimulation of the wound were similar, with a significantly greater probability of no response than LIG and CAST piglets. TRI treated piglets were least likely to respond to pin-prick with an 18G needle.

### Treatment x Time

**Fig 3:**
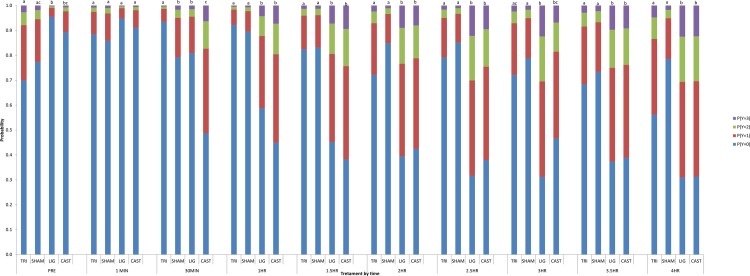
Probability of piglets of different treatments displaying responses (Y; 0 = no response, 1 = minor, 2 = moderate, 3 = severe) to stimulation over a 4 hour time period. Results combined the effect of 4g and 300g von Frey monofilaments and 18g needle for each time point. a-c Treatment groups not sharing a common letter in each time point are significantly different (P < 0.05).

There was a significant Treatment x Time interaction (P < 0.001). There was a general increase in response severity over the 4h in castrated piglets (Fig 3). At 1 min post-castration, there was no difference in the response to stimulation between treatments. TRI treated piglets were least likely to respond to stimulation from 30min to 4h post-castration, with similar responses to SHAM piglets. CAST piglets had the greatest proportion of response scores; > 1 at all time points. LIG piglets had similar responses to CAST from 1 to 4 h post-castration.

Local anesthetics have been found to reduce acute pain associated with castration in piglets when injected into the scrotum [5, 7, 8] However, they are rarely incorporated into routine husbandry procedures of commercial piggeries in many countries, including Australia. This study presents new data on the efficacy of local anaethetics applied as a topical spray for reducing post-operative wound sensitivity following castration in piglets. Application of topical anaesthetic provided amelioration of pain up to 4h post procedure for piglets undergoing surgical castration using the farmer-applied, spray-on topical anaesthetic Tri-Solfen®. These findings may have welfare implications for all piglets undergoing this and similar surgical procedures, particularly as these trials confirmed that topical anaesthetic spray can be readily incorporated into routine farm management practices.

Topical anesthesia, applied during and immediately after castration has previously been found to be practical and effective for reducing post-operative pain associated with surgical husbandry procedures in lambs [11, 12, 18, 19] and calves [14, 20].

Local anaesthetic agents impede nociception, acting directly on nerve tissue to inhibit the conduction of nerve impulses responsible for the sensation of pain. They are absorbed through mucosal surfaces and damaged skin, and can induce rapid and profound local anaesthesia when applied to open wounds [11, 12, 21–23]. Tissue damage resulting from castration results in the localized release of chemical mediators including substance P and bradykinin, invoking an inflammatory response, with vasodilation, oedema and increased nociception leading to hyperalgesia [19, 24]. These mediators can have a prolonged effect, leading to increased sensitization of neurons to nociceptive signals (hyperalgesia), and exacerbated pain to noxious stimuli. Local anaesthetics suppress bradykinin and substance P-mediated signaling, with attenuation of cutaneous micro-vascular flare responses in damaged tissue and reduced inflammation, decreasing hyperalgesia of the wound and surrounding skin.

Local anaesthesia via injection of has been shown to reduce pain accompanied by significant reductions in the cortisol response of surgical castration [5, 7]. Our findings indicate that topical anaesthetic spray causes a similar reduction in pain, with CAST piglets displaying a greater response to wound stimulation than SHAM or TRI piglets. These findings are consistent with previous observations of piglets receiving local anaesthetic infiltration of the spermatic cord and scrotum [7, 25] and with observations of absent or significantly reduced pain responses in lambs and calves treated with topical anaesthetic into castration wounds [12, 14].

In this study, piglets had the greatest response to needle pin prick stimulation of the tissue within and surrounding the wound. This was expected as this stimulus is known to induce a detectable pain response [15–17]. This method proved successful at detecting the presence or absence of anaesthesia in the wound and surrounding tissues as sham castrated piglets displayed a response to needle stimulation at all time points.

Castrated piglets that did not receive topical anesthetic had significantly greater pain responses from 2 to 4 hours following castration, demonstrating the recruitment of the pain cascade in CAST piglets not receiving any pain management. It appears likely that the pain management from the injected lignocaine in LIG piglets had diminished by the 2 h time point, as there was a rise in sensitivity from 30min to 1.5 h, and the responses of LIG piglets did not differ from CAST piglets after 2 h. This is to be expected as the lignocaine is reported to have effect for 1.5–2 hrs. In addition, it is possible that the injected lignocaine had little effect on the skin and peri-testicular tissue, leaving them vulnerable to nociception.

TRI piglets displayed apparent wound anaesthesia up to 4 h, with significantly lower pain scores than CAST and LIG piglets. The inclusion of long acting Bupivicaine in the topical anaesthetic may explain this extended duration. This indicates that topical anaesthetic spray is effective at reducing wound pain for a prolonged period following castration.

Response scores of castrated piglets were not significantly different immediately following castration (1min). It is hypothesized that this was due to the up-regulation of sensory receptors following the incision, resulting in a release of natural pain mediators including endorphins to mitigate immediate pain associated with the incision and tissue removal. From 30min following castration TRI treated piglets had the lowest pain response scores, indicating effective anaesthesia to wound stimulation for at least 4 h.

## Conclusions

These findings suggests that the global pig industries currently using piglet castration, may benefit from consideration of these findings as they offer a possible means of improving the welfare of male piglets during this routine but painful husbandry procedure.

## Supporting information

S1 File Anonymised dataset. This is the dataset used for analysis in this trial.
(XLSX)
